# Lipid peroxidation in osteoarthritis: focusing on 4-hydroxynonenal, malondialdehyde, and ferroptosis

**DOI:** 10.1038/s41420-023-01613-9

**Published:** 2023-08-29

**Authors:** Xiong Zhang, Liangcai Hou, Zhou Guo, Genchun Wang, Jingting Xu, Zehang Zheng, Kai Sun, Fengjing Guo

**Affiliations:** Department of Orthopedics, Tongji Hospital, Tongji Medical College, Huazhong University of Science and Technology, Wuhan, Hubei 430030 China

**Keywords:** Osteoarthritis, Cell death, Chronic inflammation

## Abstract

Osteoarthritis (OA) is a multifactorial and increasingly prevalent degenerative disease that affects the whole joint. The pathogenesis of OA is poorly understood and there is a lack of therapeutic interventions to reverse the pathological process of this disease. Accumulating studies have shown that the overproduction of reactive oxygen species (ROS) and ROS-induced lipid peroxidation are involved in the pathogenesis of OA. 4-Hydroxy-2-nonenal (4-HNE) and malondialdehyde (MDA) have received considerable attention for their role in cartilage degeneration and subchondral bone remodeling during OA development. Ferroptosis is a form of cell death characterized by a lack of control of membrane lipid peroxidation and recent studies have suggested that chondrocyte ferroptosis contributes to OA progression. In this review, we aim to discuss lipid peroxidation-derived 4-HNE and MDA in the progression of OA. In addition, the therapeutic potential for OA by controlling the accumulation of lipid peroxidation and inhibiting chondrocyte ferroptosis are discussed.

## Facts

- Lipid peroxidation contributes to OA progression.
- The lipid peroxidation products 4-HNE and MDA are closely associated with OA pathogenesis.
- Inflammatory mediators, ferroptosis inducers, mechanical overloading, and iron overload increase the accumulation of ROS and lipid peroxidation in chondrocytes, which can lead to chondrocytes ferroptosis and might contribute to the pathogenesis.
- Inhibiting lipid peroxidation and ferroptosis by antioxidants might provide a novel therapy for OA.

## Open questions

- What are the underlying mechanisms that lipid peroxidation is involved in the progression of OA?
- What is the specific role of 4-HNE, MDA, and ferroptosis in OA?
- What are the links between GPX4, iron homeostasis, and IL-β in chondrocyte ferroptosis?
- Are there any inducers or inhibitors that can target ferroptosis for the treatment of OA?

## Introduction

Osteoarthritis (OA) is the most common joint disease; more than 303 million people worldwide suffer from OA [1]. OA is becoming more prevalent and burdensome, especially in the elderly population [2]. The current treatment of OA involves disease education, guidance for sports activities, medication, and surgical treatment [3]. Although pharmacotherapy can alleviate or control pain and inflammation of OA, it fails to reverse the disease progression and may produce side effects during chronic administration. Joint replacement surgery has been a routine treatment for end-stage OA, but the timing of surgery is crucial because joint replacements will not survive forever [4]. There are many known risk factors for OA such as aging, gender, obesity, genetics, injury, and abnormal loading [5]. The pathogenic mechanisms caused by these risk factors are poorly understood. However, oxidative stress plays a critical role and has been highlighted in these pathologic processes of OA [6, 7].

Oxidative stress represents an abnormal condition that reactive oxygen species (ROS) production and ROS elimination are unbalanced due to an increase in ROS [8]. Excessive ROS can attack proteins, DNA, and lipids such as polyunsaturated fatty acids, which is an important component of cellular membranes [9].

Lipid peroxidation is a specific process of lipid oxidation, which received more attention for its involvement in various and numerous pathological states [10]. Lipid peroxidation includes three different mechanisms: (1) enzymatic oxidation, (2) nonenzymatic free radical-mediated oxidation, and (3) nonenzymatic free radical-independent oxidation [11]. Free radicals can attack lipids that contain carbon-carbon double bond(s), such as polyunsaturated fatty acids (PUFAs), and generate lipid peroxidation products [12]. Lipid hydroperoxides (LOOH) are primary products, which can be degenerated into secondary aldehydes such as MDA, propanal, hexanal, and 4-HNE [12]. In this review, we focused on the role of free radical-mediated peroxidation of lipids and its products in OA.

Ferroptosis is a form of recently discovered cell death, which occurs with iron and ROS dependence, characterized by lipid peroxidation [13, 14]. A growing amount of studies have demonstrated that ferroptosis participates in the progression of many different diseases such as cancer [15], Alzheimer’s disease [16], Parkinson’s disease [17], traumatic brain injury [18], rheumatoid arthritis (RA) [19], ischemia-reperfusion injury [20] and intervertebral disc degeneration [21]. Recently, our group first reported the role of chondrocyte ferroptosis in the progression of OA [22]. In addition, several studies reconfirmed chondrocyte ferroptosis existing in OA and provided some potential therapeutic strategies [23–26].

Therefore, we will review the role of ROS-induced lipid peroxidation in chondrocytes and its decomposition end products 4-HNE and MDA in OA. Moreover, we will discuss the therapeutic potential of regulating lipid peroxidation and chondrocyte ferroptosis for OA.

## Oxidative stress and lipid peroxidation

Oxidative stress is a concept for research in redox biology and medicine, and it results from the increased levels of ROS and reactive nitrogen species changing the normal redox status [27, 28]. ROS is a term that describes chemically reactive chemicals that contain oxygen [29]. Generally, ROS includes free radicals and non-radicals. Free radicals usually include superoxide radicals (O•2–), hydroxyl radicals(•OH), hydroperoxyl radicals (HOO•), alkoxy radicals (RO•), and peroxyl radicals (ROO•) [28]. The non-radicals include hydrogen peroxide (H_2_O_2_), ozone (O_3_), and nitric oxide (NO) [30]. Free radicals are very unstable (contain at least one unpaired electron in its outer orbital) and can attack biomolecules such as DNA, proteins, and lipids and cause cellular injury [30]. Among all the free radicals, O•2− is the most abundant, and •OH is the most harmful [30]. The mitochondria often act as the most source of ROS for their containing of the respiratory chain and utilizing most of the intracellular oxygen [31]. Besides, the other sources of endogenous ROS include the protoplasm (NADPH oxidases, hemoglobin, riboflavin), the endoplasmic reticulum (Cytochromes P450 and b5), the peroxisomes (oxidases, flavoproteins), and lysosomes (myeloperoxidase, metal ions) [32].

Lipid peroxidation is a typical biologically relevant free radical chain reaction [33]. Like other chain reactions, the phases of lipid peroxidation include initiation, propagation, and termination. In the initiation step, free radicals such as •OH abstract hydrogen from lipids and form lipid radicals (L•) [12]. In the propagation phase, a lipid radical reacts with oxygen, producing a lipid peroxyl radical (LOO•) that may interact with another lipid (L•), making a LOOH and another lipid radical (L•) that can repeat the cycle [34]. In the termination phase, the propagation stops by antioxidants or lipid peroxyl radicals interacting with other radicals to produce a stable nonradical.

**Fig. 1:**
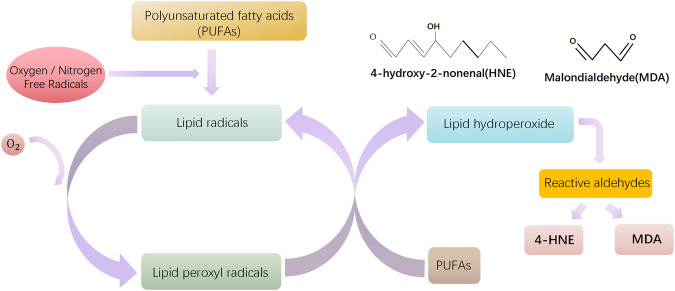
Simplified schematic illustrating the peroxidation of polyunsaturated fatty acids (PUFAs). Free radical-mediated oxidation lipid peroxidation includes three steps: initiation, propagation, and termination. In the initiation phase, ROS/ RNS free radicals react with PUFAs and abstract an allylic hydrogen thus forming lipid radicals. In the propagation phase, the lipid radicals react with oxygen and form lipid peroxyl radicals, and then lipid peroxyl radicals react with PUFAs to form new radicals and lipid hydroperoxides. In the termination phase, the propagation can be blocked by antioxidants or lipid radicals by donating a hydrogen atom to lipid peroxyl radicals resulting in the formation of nonradical products. Due to the instability of lipid hydroperoxides, they may degrade into secondary products. 4-HNE and MDA are the main products among the many reactive aldehydes.

Lipid peroxidation can generate a wide array of peroxidation products, including primary products and secondary products. LOOH are the most abundant primary products [12] and the decomposition of LOOH can generate reactive carbonyl species (RCS), such as short-chain carbonyl derivatives and oxidized truncated phospholipids [35]. RCS are more stable and toxic than free radicals because they can attack biomolecule targets (proteins, DNA, and aminophospholipids) far from their site of formation [36]. The short-chain carbonyl derivatives include a variety of lipid aldehydes, such as α, β-unsaturated aldehydes, di-aldehydes, and ketoaldehydes [35, 36]. Among these, the α, β-unsaturated aldehyde 4-hydroxy-2-nonenal (4-HNE) and di-aldehyde malondialdehyde (MDA) are most studied (Fig. 1).

The reaction between RCS and biomolecules, called lipoxidation, can generate a variety of adducts collectively called advanced lipoxidation end products (ALEs) [37]. In addition to acting as biomarkers of oxidative damage, ALEs also have a role in the pathological processes of some oxidative-based diseases [38]. Among them, 4-HNE-modified proteins [39] and MDA-modified proteins [40] are the most commonly studied.

Many studies have demonstrated that lipid peroxidation is involved in different diseases and disorders such as cardiovascular diseases [35], cancer [41], neurodegenerative diseases [42], and diabetic retinopathy [28]. In addition, lipid peroxidation products such as 4-HNE [43], MDA [44], and isoprostanes [45] are widely used as biomarkers of lipid peroxidation.

## Pathological aspects of lipid peroxidation in OA

During the past two decades, mounting studies have demonstrated that lipid peroxidation has a role in the onset and progression of OA. Clinical studies have shown that lipid peroxidation levels are increased in OA patients. A previous study reported significantly increased lipid peroxidation (measured by 4-HNE and MDA) in the synovial cells of OA than in RA patients and controls [46]. Moreover, MDA and 4-HNE protein adducts were also identified in OA tissue sections by using immunohistochemical methods [47]. As is well known, isoprostanes can serve as reliable markers of lipid peroxidation [48]. Basu et al. [49] reported that the levels of 8-isoprostaglandin F2α (one of the major isoprostanes) in serum and synovial fluid were higher in OA patients compared to control subjects. Moreover, Franz and his colleagues [50] also found increased levels of 8-isoprostane F2α in both synovial fluid and serum from the OA group. The studies suggest that lipid peroxidation is increased in OA.

### Lipid peroxidation damage in OA

Until now, the specific mechanisms of how lipid peroxidation is involved in the development of OA have not been completely clarified. Cartilage degeneration is one of the most prominent features of OA. A previous study from Tiku et al. [51] showed that chondrocyte lipid peroxidation may contribute to cartilage aging and OA. Furthermore, they demonstrated that chondrocyte lipid peroxidation contributed to the oxidation and degradation of collagen, which in turn may aggravate OA by increasing the sensibility of cartilage to mechanical fatigue [51]. Recently a study found that lipid peroxidation could regulate chondrocyte mitochondrial dynamics and cartilage injury response [52]. Animal experiments also demonstrated that lipid peroxidation has a role in the pathogenesis of the early stages of OA. Zubavlenko et al. [53] have observed the activation of the lipid peroxidation process in posttraumatic osteoarthrosis (PTOA) rat models. Moreover, Gladkova et al. [54] also found that increased production of primary and intermediate lipid peroxidation products (hydroxyl radicals, MDA) in PTOA rats. Previous studies have reported that lipid peroxidation has a role in osteoporotic bone loss [55] and synovial inflammation [56]. However, there is not much research directly examining the role of lipid peroxidation in the subchondral bone remodeling, synovial inflammation of OA. In order to introduce the role of lipid peroxidation in OA, we reviewed existing research focused on lipid peroxidation-derived products and lipid peroxidation-induced ferroptosis.

### Lipid peroxidation-derived aldehydes in OA

As the most abundant and toxic lipid peroxidation-derived aldehyde, 4-HNE has been shown to be involved in the development of many disorders such as cardiovascular diseases [57], neurological diseases [58], inflammatory bowel disease [59], diabetes [60], RA [61], and different types of cancer [62–64]. Furthermore, 4-HNE can react with macromolecules (proteins, lipids, and nucleic acids) and regulate various signaling pathways such as Nrf2, NF-κB, PKC and ERK [65]. In addition, 4-HNE has a role in numerous cell processes, including but not limited to oxidative stress [66], inflammation [67], cell proliferation [68] and cell death [69].

MDA is usually used as a biomarker for lipid peroxidation assessment [44]. Moreover, MDA can react with amino acids and yield different adducts such as MDA-lysine [40], and MDA-glycine [70]. Moreover, the MDA-acetaldehyde protein adduct termed malondialdehyde–acetaldehyde (MAA) has been reported to have a role in several diseases, like pancreatitis [71], atherosclerosis [72], RA [73], and inflammatory bowel disease [74]. Following, we will discuss the role of lipid peroxidation-derived 4-HNE and MDA in the development of OA.

#### Effects of 4-HNE on cartilage homeostasis and subchondral bone

4-HNE is one of the secondary metabolites of oxidation of PUFAs, particularly omega-6 fatty acids such as arachidonic acid and linoleic acid [75]. As the most cytotoxic aldehyde [76], 4-HNE is an α, β-unsaturated aldehyde and a highly reactive lipid-derived electrophile [77]. Studies have shown that 4-HNE and 4-HNE-modified proteins are closely associated with cartilage homeostasis by affecting the anabolic and catabolic processes of the chondrocytes. Morquette et al. [78] reported that the level of 4-HNE was increased in OA cartilage. 4-HNE could induce transcriptional and post-translational modifications of collagen II and matrix metalloproteases 13 (MMP13) [78]. At mRNA levels, 4-HNE inhibited collagen II expression but induced MMP13 expression [78]. At the post-translational level, 4-HNE accelerated collagen II degradation and activated MMP13, possibly by post-translational modification [78]. Results from another study indicated that 4-HNE-binding to collagen II could lead to multiple abnormalities of chondrocyte phenotype and function, suggesting it may contribute to interrupting the extracellular matrix (ECM) homeostasis [79].

Moreover, studies reported that 4-HNE plays a role in inflammatory responses and apoptosis of osteoarthritic chondrocytes. Vaillancourt et al. [80] reported that 4-HNE could upregulate cyclooxygenase-2 (COX-2) via activating ATF/CRE and inhibit inducible nitric oxide synthase (iNOS) via inactivating NF-κB in OA chondrocytes. They also found that 4-HNE production in OA articular tissues contributed to inflammatory responses by upregulating COX-2 expression and limiting the magnitude of transcriptional expression of iNOS [80]. Apoptosis is a form of classical programmed cell death, and chondrocyte apoptosis has been proven to have a promoting role in OA [81]. ROS-mediated oxidative damage, inflammatory stimulus, and apoptosis are all involved in cartilage degeneration and the progression of arthritis [82]. Lipid peroxidation is a process of the oxidative degradation of lipids caused by free radicals, characterized by formatting lipid peroxides, and participates in different types of cell death [83]. In their later study, Vaillancourt and his colleagues demonstrated that 4-HNE was cytotoxic and could induce apoptosis in human osteoarthritic chondrocytes [84]. Moreover, 4-HNE could suppress pro-survival Akt kinase activity yet induce Fas/CD95 and p53 expression in chondrocytes [84]. However, GSH-S-transferase A4-4 knockdown could augment 4-HNE cytotoxicity, and its overexpression could obviously inhibit 4-HNE-induced death of chondrocytes [84]. In an animal experiment, Shi et al. [85] demonstrated that carnosine could abolish 4-HNE production and attenuate cartilage degeneration in the dog model of OA. Aldehyde dehydrogenase 2 (ALDH2) is an important enzyme in the detoxification of 4-HNE [86]. Ausra et al. [87] showed that ALDH2 may play a protective role in human OA chondrocytes. Moreover, a recent study demonstrated that ALDH2 could alleviate MIA‑induced oxidative stress, inflammation and apoptosis in chondrocytes via inhibiting aquaporin 4 expression [88].

In addition, 4-HNE binding to proteins was reported to disturb the phenotype and function of chondrocytes [89]. In a recent study, Geib et al. [89] identified 4-HNE-modified proteins such as 4-HNE-modified histones, H2A and H2B, and histone deacetylase in chondrocytes of OA patients via anti-4-HNE antibodies. Moreover, Niu et al. [90] recently found that 4-HNE has a role in dampening chondrogenesis. Their results demonstrated that 4-HNE could form adducts with SOX9 and lead to its ubiquitin-mediated degradation [90].

Subchondral bone is an essential component of the joint, and the subchondral bone remodeling dominated by osteoclasts and osteoblasts can reflect the OA process [91, 92]. Data from a study showed that 4-HNE/protein adduct levels were higher in OA osteoblasts than in normal osteoblasts and when OA osteoblasts were treated with hydrogen peroxide [93]. The results showed that 4-HNE could increase osteocalcin and type I collagen synthesis while inhibiting alkaline phosphatase activity [93]. In addition, 4-HNE could change the COX-2 and interleukin-6 (IL-6) expression by affecting their production signaling pathways in osteoblasts [93]. 4-HNE inhibited IL-6 expression while inducing prostaglandin E2 (PGE2) release and COX-2 expression [93]. In human OA osteoblasts, 4-HNE could activate p38 MAPK, ATF-2/CREB and JNK1/2, but inhibit NF-κB signal [93]. Furthermore, 4-HNE modulated IL-6 and PGE2 expression via IKKα and p38 MAPK signaling pathways, respectively [93]. Thus, 4-HNE may contribute to OA development by changing the phenotypic and function of osteoblasts in OA subchondral bone.

The results mentioned above confirm that lipid peroxidation exists in OA; in addition, 4-HNE/4-HNE-modified proteins may have a valuable and attractive role in exploring the pathophysiology of OA. More studies are warranted to explore the impact of 4-HNE on cartilage homeostasis and the therapeutic potential by scavenging 4-HNE in OA.

#### Effects of MDA on cartilage collagen oxidation

MDA is another main second product of lipid peroxidation, with the formula of CH2(CHO)2, usually used as a marker of oxidative stress [94]. Tiku et al. [95] demonstrated that MDA mediates the oxidation of cartilage collagens. A previous study found that serum MDA concentration and serum C2C concentration (a marker of collagen type II degradation) were significantly increased in ACLT-induced OA dogs [96]. Moreover, the levels of MDA and C2C continuously increased during the whole process of the experiment, and MDA levels had a high positive correlation with C2C [96]. Watari et al. [97] observed that the serum levels of MDA, c-telopeptide degradation products of type II collagen (CTX-II), carboxy propeptide of type II collagen (CPII) were higher in the STR/Ort (Str) mice than in CBA mice. They also found that the level of MDA was correlated with CTX-II, but not with CPII [97].

Several studies reported that the levels of MDA in plasma were markedly higher in OA patients than in healthy controls [98–100]. Furthermore, results showed that the MDA concentrations in synovial fluid were higher compared to paired plasma samples [98]. Contrarily, a more recent study reported that there was no difference in MDA concentrations in erythrocytes and blood plasma between healthy subjects and OA patients [101]. Reasons for the different results may include the limited sample sizes, the assays for detection, different stages of the disease, and the difference between local and systemic states of oxidative stress. The association between MDA concentrations in plasma and synovial fluid in OA patients warrants further investigation. More clinical trials with larger sample sizes and combinations with other lipid peroxidation biomarkers would help to draw more solid conclusions.

As is a highly reactive aldehyde, MDA can combine acetaldehyde (AcA) to form MAA or the MAA adduct [102]. AcA is derived from exogenous sources or the breakdown of MDA [103]. MAA-adducts that are derived from the reaction of MAA and proteins have been proven to be immunogenic and possess proinflammatory and profibrogenic properties [104]. Moreover, studies have demonstrated that MAA-adducts may play a role in several diseases, especially alcoholic liver injury and atherosclerosis [104, 105]. In addition, MDA modifications and anti-MDA-modified protein autoreactivity have been reported to play a role in RA [106]. In RA, MDA-related antigens and MDA reactive antibodies may have a role in the synovial pathogenesis of RA [106]. Moreover, hypermutated anti-MDA IgG clones could enhance osteoclastogenesis and may lead to joint destruction [106]. In a more recent study, Sakuraba et al. [107] also found that anti-MDA antibodies have a role in accelerating bone erosion in RA via inducing glycolysis and lipid biosynthesis of osteoclasts. Furthermore, methotrexate (MTX), the immunosuppressant commonly used for the treatment of RA, has been demonstrated that could reduce inflammation and subsequent tissue damage by inhibiting MAA-adducts formation and scavenging superoxide in RA [108].

However, studies focused on the role of MAA-adducts and anti-MDA antibodies in OA are lacking. In a previous study by Mikuls et al. [102], the serum levels of IgA anti-MAA and IgG anti-MAA were higher than health controls. Up to now, the research focus of MAA and MAA-adducts has been mainly on inflammatory diseases or autoimmune diseases. Nevertheless, with more and more in-depth research on lipid peroxidation in OA, the roles of MDA, MAA, or MAA-adducts may become increasingly important.

### Lipid peroxidation-driven chondrocyte ferroptosis in OA

Ferroptosis was first reported by Dixon et al. [13] in 2012, and they identified it as a unique form of cell death caused by iron overload and lipid peroxidation, different from apoptosis and autophagy. The inactivation of antioxidant systems such as glutathione peroxidase 4 (GPX4), glutathione (GSH), and coenzyme Q10 (CoQ10) system promotes ferroptosis [109]. Ferroptosis is characterized by ROS generation, GPX4 depletion, LOOH accumulation, and iron overload [14]. Recent studies have demonstrated that these features, which can initiate ferroptosis, are accompanied by the pathogenesis of OA [22, 23, 110].

Our group first demonstrated that chondrocyte ferroptosis have an important role in the progression of OA. We found that both interleukin-1 beta (IL-1β) and ferric ammonium citrate (FAC) could induce ROS, lipid ROS accumulation, and ferroptosis-related proteins (GPX4, ACSL4, P53, and SLC7A11) expression changes in chondrocytes [22]. Following our study, many investigations have shown the involvement of chondrocyte ferroptosis in the development of OA and provided potential therapeutic strategies targeting lipid peroxidation and ferroptosis [23, 24, 111]. These studies are summarized in Table 1.

**Table 1:** Regulation of lipid peroxidation as a potential therapeutic strategy for OA.

Regulators	Tissue (cell) type	Effect	Reference
Ferrostatin‐1	Mouse chondrocytes Mouse cartilage	Attenuates the IL-1β-induced cytotoxicity, ROS, and lipid ROS accumulation Rescues GPX4 and SLC7A11 expression Attenuates P53 and ACSL4 expression Attenuates cartilage degradation and increases collagen II and GPX4 expression in mouse OA model	[22]
	Mouse chondrocytes Mouse cartilage	Rescues TBHP-induced ferroptosis Reduces lipid peroxidation Decreases iron accumulation maintains GPX4/GSH function Rescues TBHP-induced decrease of mitochondrial membrane potential (MMP) Reverses TBHP-induced decrease of GPX4 and Fth1 decreases ACSL4 and PTGS2 Ameliorates ACLT-induced bone volume fraction increasing and SBP thickening restricts osteophyte formation Protects the rupture of outer mitochondrial membrane (OMM) of chondrocytes in the ACLT-induced OA mouse mode	[23]
Vitamin E	Rabbits chondrocytes	Diminishes the cartilage matrix degradation and release by reducing levels of lipid peroxidation activity	[70]
Deferoxamine	Mouse chondrocytes Mouse cartilage	Rescues TBHP-induced ferroptosis Reduces lipid peroxidation decreases iron accumulation maintains GPX4/GSH function Rescues TBHP-induced decrease of mitochondrial membrane potential (MMP) Reverses TBHP-induced decrease of GPX4 and Fth1 decreases ACSL4 and PTGS2 Ameliorates ACLT-induced bone volume fraction increasing and SBP thickening restricts osteophyte formation Protects the rupture of the outer mitochondrial membrane (OMM) of chondrocytes in the ACLT-induced OA mouse model	[23]
		Alleviates IL-1β- induced and erastin-induced cytotoxicity, abrogates ROS and lipid ROS accumulation and the increase in MDA Promotes Nrf2 antioxidant system activation Alleviates OA progression in DMM mice model	[133]
CoQ10	GPX4-CKO mouse	Alleviates osteophyte formation in the DMM model Protects against cartilage degeneration Protects against cartilage degeneration in GPX4-CKO mice	[114]
Stigmasterol	Mouse chondrocytes	Reduces IL-1β-induced ferroptosis via SREBF2	[26]
Naringenin	Mouse chondrocytes Mouse cartilage	Reduces iron overload-induced ROS, lipid hydroperoxide, and MDA Alleviates cartilage damage under iron overload	[124]
Astaxanthin	Rat chondrocytes	Inhibits IL-1β-induced chondrocyte ferroptosis Attenuates cartilage damage in OA model rats	[127]
Metformin	Mouse chondrocytes Mouse cartilage	Attenuates Erastin-induced ferroptosis in mouse chondrocytes	[25]
Biochanin A	Mouse chondrocytes Mouse cartilage	Attenuates Erastin-induced cartilage degeneration protects chondrocytes from iron overload damage Reduces the severity of OA induced by iron overload	[134]

#### GPX4-dependent chondrocyte ferroptosis in OA

GPX4 has recently attracted more attention in OA due to its ability to eliminate lipid peroxides and inhibit ferroptosis [23]. System Xc−/GSH/GPX4 pathway is the most studied pathway in OA. Miao et al. [23] found that GPX4 could regulate ferroptosis in chondrocytes; on the other hand, GPX4 could regulate ECM degradation via MAPK/NF-κB signaling pathway [23]. The results enrich research on GPX4 and chondrocyte ferroptosis and provide a new idea for OA treatment.

Moreover, recent studies have demonstrated that IL-1β treatment could promote ferroptosis and decrease the level of GPX4 protein in chondrocytes [22, 112]. Lv and his colleagues reported that the RNA-binding protein SND1 promoted ferroptosis in IL-1β-treated chondrocytes by affecting the HSPA5-GPX4 axis [112]. The results provide new insights into the mechanisms by which IL-1β induces chondrocyte ferroptosis.

In another study, researchers found that transient receptor potential vanilloid 1 (TRPV1) could prevent chondrocyte ferroptosis by upregulating the expression of GPX4 [113]. In the primary chondrocytes, Trpv1 activation could restore TBHP-induced Gpx4 decrease, while Trpv1 activation substantially restored.

**Fig. 2:**
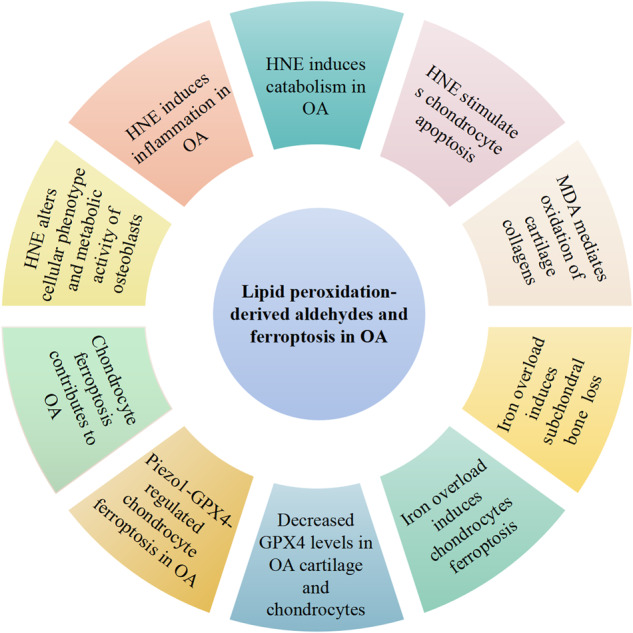
Lipid peroxidation association with osteoarthritis. OA osteoarthritis, 4-HNE 4-hydroxy-2-noneal, MDA malondialdehyde, GPX4 glutathione peroxidase 4. Association of lipid peroxidation and its secondary products 4-HNE and MDA with OA, cartilage catabolism and inflammation, subchondral bone metabolism, chondrocyte apoptosis, chondrocyte ferroptosis.

As is well known, exposure of chondrocytes to excessive mechanical loading accelerates the development of OA [114]. Piezo-type mechanosensitive ion channel component 1 (Piezo1) is one of the piezo channels present on the cell membrane and works as a mechanotransducer [115]. A recent study reported that mechanical overloading could induce GPX4-regulated chondrocyte ferroptosis in OA Piezo1 channel facilitated calcium influx [111]. Ferroptosis suppressor protein (FSP-1) and coenzyme Q10 (CoQ10) treatment could attenuate the progression of OA in GPX4-deficient mice [111]. Taken together, all results observed in mentioned studies indicate a new underlying mechanism of mechanical overloading to the progression of OA, that is, the GPX4-associated ferroptosis pathway (Fig. 2).

#### GPX4-independent chondrocyte ferroptosis in OA

Iron homeostasis is essential for joint health, and iron overload in joints has proved to be strongly associated with the pathogenesis of OA [110]. Several studies have demonstrated that iron overload can cause oxidative stress damage and induce apoptosis [116] in chondrocytes. Jing et al. [117] demonstrated that iron overload could cause cartilage degeneration by inducing oxidative stress and mitochondrial dysfunction [117]. In addition, FAC treatment could greatly promote the expression apoptosis marker of cleaved caspase‑3 in chondrocytes [118]. In another study, Karim et al. [116] also found that high doses of FAC treatment induced the generation of ROS, inhibited collagen II syntheses, and facilitated the apoptosis of chondrocytes.

Excessive iron ions can induce membrane lipid peroxidation via Fenton reaction and cell damage. FAC has been shown to have a role in inducing ferroptosis in chondrocytes in our previous study [22]. We found that FAC could significantly increase the level of ROS and lipid ROS in chondrocytes [22]. Moreover, FAC treatment promoted the expression of P53 and ACSL4 while inhibiting GPX4 and SLC7A11 in chondrocytes [22]. In addition, Pan et al. [119] found that FAC and IL-1β treatment elevated levels of ROS and LPO in chondrocytes. Not only that, the levels of LPO biomarker MDA were greatly increased in chondrocytes treated with FAC and IL-1β [119].

IL-1β is a commonly used proinflammatory cytokine to establish in vitro model of OA [120]. The ability of IL-1β to induce chondrocyte catabolism [121] has been well known for a long time, but it is only recently that IL-1β has been shown to disrupt chondrocyte iron homeostasis [117] and trigger chondrocyte ferroptosis [22, 122]. Jing et al. [117] found IL- 1β could promote iron influx and cause chondrocytes iron overload. Meanwhile, excess iron could increase matrix metalloproteinases (MMPs) expression induced by IL- 1β [117]. Moreover, several studies recently reported the role of IL- 1β in inducing ferroptosis in chondrocytes [26, 119, 122]. Although IL-1β-mediated chondrocyte ferroptosis was observed in the aforementioned studies, the specific mechanisms are unclear. In addition to increased ROS and lipid ROS [122], the levels of intracellular iron can also be augmented after IL- 1β treatment in chondrocytes. Mo et al. [26] found that the concentration of Fe^2+^ increased in ATDC5 cells after stimulation with IL‐1β. In addition, Wang et al. [122] observed that IL-1β could increase the intracellular iron and mitochondrial iron levels in chondrocytes. Recently, we have demonstrated nuclear receptor coactivator 4 (NCOA4) could aggravate OA by promoting chondrocyte ferroptosis and ferritinophagy [123]. We found IL-1β could disturb chondrocyte iron homeostasis via JNK-CJUN-NCOA4 [123]. The role of NCOA4-mediated ferritinophagy in chondrocytes gives us further insight into the connection between IL-1β and ferroptosis in OA pathogenesis. Thus, IL-1β induced iron metabolism disorders may be responsible for chondrocyte ferroptosis and cartilage degradation in OA cartilage.

As an effective iron chelator, deferoxamine (DFO) can chelate free iron and is used to relieve the damage of iron overload. Previous research demonstrated that DFO could strongly prevent the formation of free radicals caused by IL-1α treatment in synovial fluid of temporomandibular joint arthritis in rats [124]. Another study showed that DFO could suppress excessive collagenase-mediated type II collagen cleavage and protease, production of cytokine, and COL10A1 expression while upregulating adenosine monophosphate-activated protein kinase and Krebs cycle genes in human osteoarthritic cartilage [125]. In more recent studies, researchers found that DFO could promote recovery of traumatic spinal cord injury [126] and slow the process of disc degeneration [21] by inhibiting ferroptosis in animal models. Moreover, Lin et al. [127] also found that DFO could alleviate iron overload-induced ECM degradation in FVIII-deficient hemophilic mice. In addition, our group and other researchers have recently demonstrated that DFO could alleviate OA by inhibiting chondrocyte ferroptosis [23, 128]. More details about DFO are summarized in Table 1.

### Lipid peroxidation and ferroptosis promote bone loss in subchondral bone

The modification of subchondral bone, including early-stage bone loss, late-stage bone sclerosis and histopathological alterations, plays an important role in the pathogenesis of OA [129]. Recently studies have reported the role of iron accumulation and ferroptosis in bone loss. He et al. [130] recently find that subchondral bone loss in the iron-overload-induced model of knee OA. The mechanisms of iron-overload-induced bone loss mainly include the increased osteoclastogenesis and bone resorption capacity of osteoclasts [131], lipid peroxidation accumulation and ferroptosis in osteoblasts [132]. Moreover, studies have demonstrated that iron chelator DFO [133] and antioxidant N-acetylcysteine (NAC) [130] could save bone loss induced by iron accumulation. These results suggest that ferroptosis have a role in OA subchondral bone homeostasis and structural integrity. Therefore, targeting iron metabolism disorders, lipid peroxidation accumulation, and osteoblast ferroptosis in subchondral bone may provide new therapeutic strategies for OA.

### The role of ferroptosis in osteoarthritis synovial membrane

As a degenerative disease of the whole joint, OA affects articular and periarticular tissues such as synovium. Moreover, synovial inflammation is a critical OA-related pathological change. Previous studies have reported that iron overload could promote synovial inflammation and synovial hyperplasia in joint diseases such as hemophilic arthropathy [110]. Although increasing attention has been given to the role of ferroptosis in OA to date, most researchers pay more attention to chondrocyte ferroptosis and cartilage degeneration rather than synovitis. Until recently, efforts in synoviocyte ferroptosis research have focused mainly on RA [134]. However, some investigators started exploring the role of ferroptosis in the synovial tissue of OA [135, 136]. By using bioinformatics analysis Xia et al. identified and verified ferroptosis-related genes (ATF3, IL-6, IL-1B and EGR1) in the synovial tissue of OA, which were possibly associated with synovial hyperplasia [135]. Notably, in addition to synoviocytes, synovial macrophages also have an important role in synovial inflammation of OA [137]. Therefore, future studies could focus on the role of synoviocytes and macrophages ferroptosis in OA synovial membrane, which may provide a new direction for future studies on OA.

## Antioxidants

Antioxidants are reducing substances that inhibit oxidation and reduce the occurrence of multiple disorders [138]. The above studies have demonstrated the critical role of excessive ROS and lipid ROS in OA progression. Therefore, antioxidants may be valuable therapeutics for OA.

### Enzymatic antioxidants

The antioxidative defense grid of the human body includes an array of enzymatic and nonenzymatic antioxidants [139]. The enzymatic antioxidative defense system commonly includes superoxide dismutases (SODs), catalase (CAT), and GPX. In addition, the aldehyde dehydrogenases and GSH-S-transferase are also important aldehydes detoxifying enzymes [86].

SODs are metalloenzymes that require a metal cofactor for their activity. By catalyzing the conversion of superoxide anion to hydrogen peroxide and oxygen, SODs can eliminate the damage of ROS. A previous study reported the levels of SOD family members (SOD1, SOD2, and SOD3) were drastically lower in OA cartilage than in macroscopically normal cartilage, especially SOD2 [140]. Depletion of SOD2 in chondrocytes gave rise to increased ROS levels yet decreased collagenase expression [140]. Besides, the decrease in the SOD activity in end-stage osteoarthritic synovium and cartilage was also reported in a recent study [141]. These studies showed that the total SOD activity was decreased and SOD down-regulation in cartilage may be involved in the development of OA. However, whether SOD can be a useful biomarker or a therapeutic target for OA are unknown.

Catalases can protect cells from oxidative damage by degrading hydrogen peroxide to water and oxygen (2H_2_O_2_ --> 2 H_2_O + O_2_) [142]. CAT is one of the antioxidant enzymes which can protect against free radical attack [139]. Researchers found that expression of mitochondrially targeted CAT promoted Akt phosphorylation and attenuated phosphorylation of proapoptotic signaling proteins under conditions of oxidative stress in human articular chondrocytes [143]. In addition, transgenic mice that express human catalase targeted to the mitochondria exhibited less severity of age-related OA compared to WT mice [143]. A study reported that CAT activity was markedly higher in OA cartilage compared to the healthy control group [144]. As an important antioxidant enzyme, CAT has a role in protecting chondrocytes by reducing TNF-α-induced apoptosis [145]. The CAT activity increase in OA patients may attribute to the compensating protective role of CAT.

GPXs can catalyze the reduction of hydrogen peroxide or organic hydroperoxides to water or corresponding alcohols with the help of GSH [146]. Mammalians possess four major selenium-dependent GPX isozymes (GPX1, GPX2, GPX3, and GPX4) that can protect against oxidative stress [147]. A previous study reported that activities of antioxidant enzymes such as SOD, CAT, GPX, GSH reductase and glutathione-S-transferase were significantly increased in knee OA synovial fluid [148]. GPX1 has a role in various cellular processes, such as anti-oxidation, anti-apoptosis, and regulation of cell differentiation [149]. Results showed that the expression of GPX1 considerably decreased in OA tissues and cells [149]. Meanwhile, GPX1 overexpression constrained oxidative stress and apoptosis in OA chondrocytes by regulating CREB/HO-1 [149]. GPX4 is an important enzyme that can reduce esterified phospholipid hydroperoxides and regulate ferroptosis [150]. Zhang et al. [151] demonstrated that homocysteine could enhance GPX4 methylation, thus inducing ferroptosis in the nucleus pulposus. Moreover, they found that folic acid could repress ferroptosis in nucleus pulposus cells by downregulating the methylation level of GPX4 [151]. Moreover, as a key regulator of ferroptosis, GPX4 and its role in OA have been demonstrated in three recently published studies [22, 23, 111]. First of all, GPX4 expression in the damaged cartilage of OA patients was markedly decreased compared to in undamaged cartilage [23]. Secondly, the key inflammatory cytokine in OA IL-1β could suppress the expression of GPX4 in chondrocytes [22]. Thirdly, GPX4 levels decreased in cartilage from the OA animal models, and knockout of GPX4 exacerbated mice experimental OA process [111]. Taken together, the protective role of GPX4 in OA is worth an in-depth study and may provide a novel insight into the molecular mechanism and potential treatment of OA.

### Nonenzymatic antioxidants

The nonenzymatic antioxidant system consists of GSH, vitamin C, vitamin, and CoQ10. As a natural tripeptide, GSH consists of glutamic acid, cysteine and glycine, and it mainly exists in the forms of reduced GSH and oxidized glutathione (glutathione disulfide (GSSG)), and glutathione-protein mixed disulfides [152, 153]. GSH can prevent lipid peroxidation caused by toxic oxygen radicals by reducing the endogenously produced hydrogen peroxide with the help of GPX. In this process, the oxidation and reduction process between GSH and GSSG forms a redox cycle [154]. Moreover, GSH can detoxify 4-HNE by forming GS-4-HNE adducts or serving as a cofactor for alpha-class glutathione transferases (GSTAs) [155].

In OA, GSH was reported to enhance the antioxidative capacity of hyaluronic acid and modulate the expression of proinflammatory cytokines in human fibroblast-like synoviocytes [156]. Furthermore, GSH has a role in augmenting the effect caused by hyaluronic acid on OA patients [156]. Moreover, Zhu et al. [157] reported that GSH can act as a mediator of cartilage oxidative stress resistance and resilience during aging and OA. They found different stressors such as aging, inflammation, biomechanical loading, and pro-oxidant stimuli could affect GSH content and redox balance in experimental models of OA-related stress [157]. In addition, a recently published review introduced the protective role of GSH and its precursor NAC in OA [158]. Beyond that, as the reducing substrate of GPX4 activity, GSH plays an essential in inhibiting ferroptosis [159]. Therefore, regulation of GSH levels may be an effective approach to inhibit chondrocyte ferroptosis and may be one of the therapeutic strategies for OA.

Vitamin C and Vitamin E are both important nonenzymatic antioxidants that help reduce free radical damage, and act as nutritional supplements for OA treatment. A previous study reported that vitamin C could prevent monosodium iodoacetate (MIA) induced changes such as cell growth inhibiting and oxidative stress increasing, apoptosis, MMPs increasing, and proteoglycan loss in a chondrosarcoma cell line (SW1353) [160]. In addition, supplement with vitamin C could mitigate MIA-induced OA in rats [160]. Results from another study demonstrated that vitamin C could attenuate senescence of human osteoarthritic osteoblasts [161].

Vitamin E is a lipid-soluble essential micronutrient with a powerful antioxidant effect. A study reported the synovial fluid vitamin E concentration considerably decreased in OA patients [162]. Another research reported that Vitamin E could protect the cartilage matrix by preventing chondrocyte lipid peroxidation [51]. Furthermore, Vitamin E protected rat mesenchymal stem cells (MSCs) against hydrogen peroxide-induced oxidative stress in vitro and improved the therapeutic potential of MSCs in the surgically-induced rat model of OA [163]. Another study reported that Vitamin E ameliorated alterations to the cartilage of knee joints induced by monoiodoacetate and diabetes mellitus in rats, which manifested as inhibition of biomarkers of inflammation [164]. A clinical trial also reported a positive effect of Vitamin E in improving clinical symptoms and reducing oxidative stress conditions in patients with late-stage knee OA [165]. Interestingly, vitamin E could also prevent lipid peroxidation and inhibit ferroptosis [166, 167].

Although much research has reported the therapeutic value of Vitamin C or Vitamin E in preventing OA, negative results [168, 169] were also reported. However, more randomized placebo-controlled trials are desperately needed to verify the therapeutic efficiency of Vitamin C or Vitamin E for OA treatment.

**Fig. 3:**
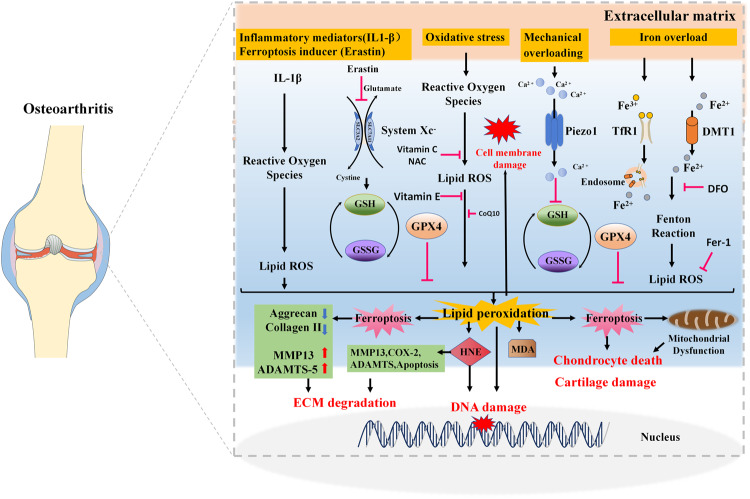
The role of lipid peroxidation in the progression of osteoarthritis. Extracellular stimuli such as inflammatory mediators, ferroptosis inducers, oxidative stress, mechanical overloading, or iron overload can elevate ROS levels and lipid peroxidation in chondrocytes and can ultimately contribute to OA progression. Inflammatory mediator IL-1β can increase the levels of ROS, lipid ROS, and the lipid peroxidation marker MDA in chondrocytes. Classical ferroptosis inducer erastin can induce chondrocyte ferroptosis by inhibiting systemic Xc-. Moreover, mechanical overloading can promote ferroptosis through Piezo1 activation and subsequent calcium influx in chondrocytes. Accumulated iron in chondrocytes can catalyze the formation of ROS, which further forms the lipid radical and leads to lipid peroxidation. Chondrocyte ferroptosis and lipid peroxidation end product 4-HNE can cause ECM degradation by breaking the equilibrium between synthesis and breakdown of extracellular matrix. Antioxidants including vitamin C, vitamin E, NAC, and CoQ10 can reduce lipid peroxidation and the formation of ROS. Iron chelator DFO can inhibit erastin-induced articular chondrocyte death, and delay articular cartilage degradation and OA progression. And the ferroptosis inhibitor Fer-1 can rescue the IL-1β–induced decrease in collagen II and increase in MMP13 expression. Antioxidant enzyme GPX4 can prevent chondrocyte ferroptosis by combating lipid peroxidation.

CoQ10 is a crucial component of the electron transport chain in mitochondria and participates in cellular energy metabolism [170]. CoQ10 is a powerful antioxidant that inhibits lipid peroxidation and scavenges free radicals [171–173], and it also shows anti-inflammatory effects [174]. It has been reported that combined administration of CoQ10 and MTX suppressed adjuvant-induced arthritis progression in rats more effectively than did MTX alone [175]. CoQ10 could potentiate both the antiarthritic and the antioxidative effect of MTX, evident by the decreased levels of protein carbonyls in plasma and levels of 4-HNE adducts and MDA adducts to plasma proteins [175]. Moreover, results from a study showed that CoQ10 could improve pain and cartilage degradation in MIA-induced OA rats [176]. In a more recent study, researchers encapsulated CoQ10 into micelles and found that CoQ10-micelles had a better chondroprotective effect than CoQ10 in OA rats [177]. Moreover, CoQ10-micelles could decrease the expression levels of catabolic and necroptotic factors in human OA chondrocytes [177]. Another study also reported the anticatabolic and cartilage protective potential in rat chondrocytes [178]. In addition, CoQ10 is essential for the health of nearly all human tissues, especially for organs with high energy demands, such as skeletal muscle [179]. A clinical study showed that CoQ10 level was positively associated with antioxidant capacity, muscle mass, muscle strength and muscle endurance in OA patients [180]. Raising the level of CoQ10 in OA patients may improve their antioxidant capacity and muscle function [180]. CoQ10 is not only an essential scavenger of ROS but is also involved in the FSP1-CoQ10-NAD(P)H pathway that has recently been reported to inhibit ferroptosis [181, 182]. Thus, based on the above evidence, CoQ10 might represent a new therapeutic modality for OA (Fig. 3).

## Conclusions and perspectives

Lipid peroxidation plays an important role in the development of OA. 4-HNE and MDA not only act as the main biomarkers for lipid peroxidation assessment and contribute to multiple pathological processes during OA development. The role of 4-4-HNE in cartilage catabolism, chondrocyte apoptosis, inflammation, and osteoclasts activity alteration suggests that 4-HNE may be a potential target in OA. Moreover, recently identified 4-HNE-modified proteins in OA chondrocytes may provide a unique opportunity to investigate how chondrocytes adapt to oxidative stress and lipid peroxidation. Not only that, 4-HNE detoxification pathways such as GSTA4-4, ALDH2, GSTs, and GSH may open new avenues to explore novel therapy for OA. MDA may be a good marker in OA but more research will be needed to confirm the levels of it in joint fluid and reveal the relationship between articular fluid MDA concentration and serum MDA concentration. The pathophysiological role of MAA and MAA-adducts in OA may be an intriguing line of investigation for future studies. In addition, growing evidence suggests that lipid peroxidation-induced ferroptosis has an important role in cartilage degradation, subchondral bone remodeling and synovitis of OA. Therefore, inhibiting lipid peroxidation and ferroptosis in chondrocytes might provide a novel therapy for OA.
